# Transcriptome Profiling in Leaves of Wheat Genotype under Heat Stress

**DOI:** 10.3390/plants11223100

**Published:** 2022-11-15

**Authors:** Kavita Lamba, Mukesh Kumar, Vikram Singh, Lakshmi Chaudhary, Rajat Sharma, Samita Yadav, Shikha Yashveer, Mohinder Singh Dalal, Vijeta Gupta, Shreya Nagpal, Manuj Saini, Navreet Kaur Rai, Rutuparna Pati, Karuna Malhotra

**Affiliations:** 1 Department of Genetics & Plant Breeding, Chaudhary Charan Singh Haryana Agricultural University, Hisar 125 004, India; 2 Department of Molecular Biology, Biotechnology and Bioinformatics, Chaudhary Charan Singh Haryana Agricultural University, Hisar 125 004, India; 3 Indian Institute of Wheat and Barley Research, Karnal 132 001, India

**Keywords:** transcriptome, wheat, heat stress responsive genes, gene regulation

## Abstract

Hexaploid wheat is the main cereal food crop for most people but it is highly influenced by climatic variations. The influence of these climatic variations was studies in wheat genotype WH -1184 in field conditions under two different environments (normal and late sown) and it was found that the genotype is less yielding under late sown conditions. To study the effects of heat stress at transcript level, it was grown under two different conditions (WH-1184 control and heat treated) in pots and transcriptome analysis based on Illumina Novoseq 6000 was carried out for the identification of the differentially expressed genes (DEGs) and metabolic processes or gene regulations influenced by heat stress which lead to a reduction in both quality and quantity of wheat production. These DEGs were utilized to set up a subsequent unigene assembly and GO analysis was performed using unigenes to analyze functions of DEGs which were classified into three main domains, i.e., biological process, cellular component, and molecular function. KEGG (Kyoto Encyclopedia of Genes and Genomes) ontology was used to visualize the physiological processes or to identify KEGG pathways that provide plants their ability to shield in adverse conditions of heat stress. From KEGG ontology, it was reported that genes which encoded protein detoxification and ABC1 domain-containing protein were upregulated while genes thatencoded glutathione transferase (GST), peroxidase, and chitinase enzymes were downregulated. Downregulation of these enzymes during heat stress causes oxidative damages in plants while upregulated proteins play a main role in detoxification to protect plants from heat stress. It was hypothesized that the yield of WH-1184 decreased 44% under heat stress due to the downregulation of genes that encoded GST, peroxidase, and chitinase enzymes which can protect plants from oxidative damage. Hence, upregulation of these genes might be helpful for the adaptation of this genotype under heat stress condition.

## 1. Introduction

Hexaploid bread wheat (*Triticum aestivum* L.) belongs to the Poaceae family and is the second-most grown, regularly fed crop in the world. However, due to the elevatedglobal temperature, the estimated worldwide demand for wheat in coming years cannot be met with the current declining production output [1,2]. According to controlled environment research, wheat grain yield is more influenced by heat stress as compared to drought stress as it decreased by 4% or a yield loss of 190 kg/ha for every degree rise in temperature over 15 °C [3,4,5]. Numerous studies have demonstrated that heat stress at booting, anthesis, and grain filling stage negatively impacts several genetic and metabolic processes within cereal plants, all of which lead to decreased grain production. High temperature also alters a number of cellular processes, damages cells, cell organelles, and the transport of photosynthates, which primarily affects photosynthesis, and as a result reduces yield. In severe circumstances, heat stress may even result in the death of plant species [6,7]. Heat tolerance is controlled by multigenes encoded various molecular processes, so the identification of genesencodingmolecular processes at the transcriptional level may be more important and advantageous for the evolution of heat tolerant wheat genotypes [8,9]. Inadequate knowledge exists on heat responsive genes and enzymes associated to heat tolerance pathway, despite recent advances in research on understanding the mechanism of heat tolerance in model plants [10]. To find and describe the differentially expressed genes (DEGs) during heat stress in various model plants, plant omics and modern biotechnologies including highly scalable sequence analysis and microarray analysis were most favored techniques.

With the advancement in next-generation sequencing (NGS), it is now the preferred technique used for studying the transcriptome, regulatory pathways, and to detect significantly expressed genes with their encoded proteins under various stresses [11,12]. RNA-Sequencing (RNA-Seq) is the better approach used to identify genome, de novo transcriptome, sequencing of short segments of cDNAs to generate contigs, study relative expression, 5′ and 3′ ends editing of annotated genes, and toperform exons and introns identification of functional genes [13,14]. In wheat, this approach has been mostly utilized to discover new and conserved stress associated genes and proteins. Azameti et al. [15] used transcriptome sequencing in wheat genotype Raj 3765 to identify differentially expressed genes (DEGs), molecular mechanisms, as well as transcriptome changes of genes and candidate genes responsible for wheat thermotolerance.

In this study, we have selected wheat genotype WH-1184 to observe the effect of heat stress at transcript level also as this genotype was found heat susceptible on evaluation of the field data (relative stress injury, membrane stability index and grain yield per plot) under normal and heat stress conditions. For further examine comprehensive knowledge of molecular processes in this genotype under control and heat stress conditions, we have used RNA- sequencing based onIllumina Novoseq 6000 to identify total differential expressed genes (DEGs) andupregulated and downregulated genes. These identified DEGs, were used for GO to analyze their functions and for KEGG ontology to analyse physiological processes of the genotype. Thus, our findings contribute tothe understanding of differences in transcriptional changes in wheat genotype studied under controlled and stressed conditions.

## 2. Results

### 2.1. Selection of WH-1184

Wheat genotype WH-1184 was selected for comparative transcriptome studies based on its percent reduction in relative stress injury, membrane stability index and grain yield per plot under different environmental conditions (normal and late sown) (Table 1). This genotype was reported as less heat tolerant at field conditions as its cells were more injured, membrane stability index and grain yield per plot were also reduced during stressed condition as compared to normal conditions. This had shown a percent reduction of −46.36, 9.37 and 38.82 percent in relative stress injury, membrane stability index, and grain yield per plot, respectively, and statistically grain yield was found significantly correlated with both parameters (RSI and MSI) under both the conditions. Thus, the genes upregulated or downregulated due to heat stress werealso observed using transcriptome analysis.

### 2.2. RNA Sequencing and QC Check of Raw Reads

On average, both samples had 67.40 million reads, of which 11.72 million were mapped. WH-1184 treated had higher GC content (55.0%) as compared to WH-1184 control (52.5%) in which percentage of Q20 (1% chance of error and 99% confidence) was found more than 99% whereas, Q30 (0.1% chance of error and 99.9% confidence) was >85% (Table 2). The results indicated that all the data qualified for downstream analysis. GC pairs are generally more stable than AT pairs; GC-rich genomes were proposed to be more adapted to high temperatures than AT-rich genomes. Previous studies consistently showed positive correlations between the GC contents of structural RNA genes and heat stress. Thermal adaptation is one possible explanation for the positive association.

### 2.3. Alignment and Expression Analysis

WH-1184 heat stressed had higher 13.8 million mapped reads (73.98%) while WH-1184 control had approximately 9.62 M (70.15%) mapped reads. The rates of uniquely mapped reads of samples, WH-1184-Control and WH-1184-Stressed, were 39.85% and 40.91%, respectively. WH-1184-Stressed plant contained more unique reads as well as unmapped reads (4.86 M) than WH-1184 control onto the reference genome, as shown in Table 3.

Expression values of genes were obtained as read counts using feature Counts software. Expressed gene was considered if it had at least one mapped read. Total numbers of expressed genes are presented in Table 4. Number of expressed gene was higher in heat stress sample. WH-1184-Heat stressed had higher number of expressed genes (87,694) while WH-1184 control had less number (76,012).The increased number of expressed genes under heat stress showed how genotypes try to adapt withexposure tohigh temperature.

### 2.4. Differential Expression of Genes

Differential expression analysis was carried out using the DESeq2 package. Genes with less than total 5 reads were removed from further analysis. Test samples (treated) were compared to the control samples.

**Figure 1:**
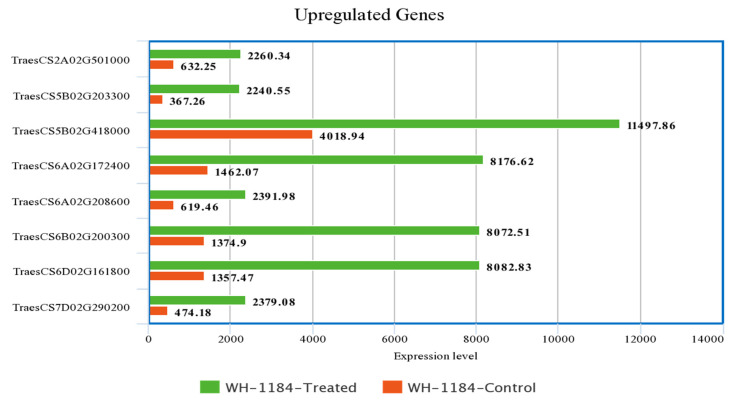

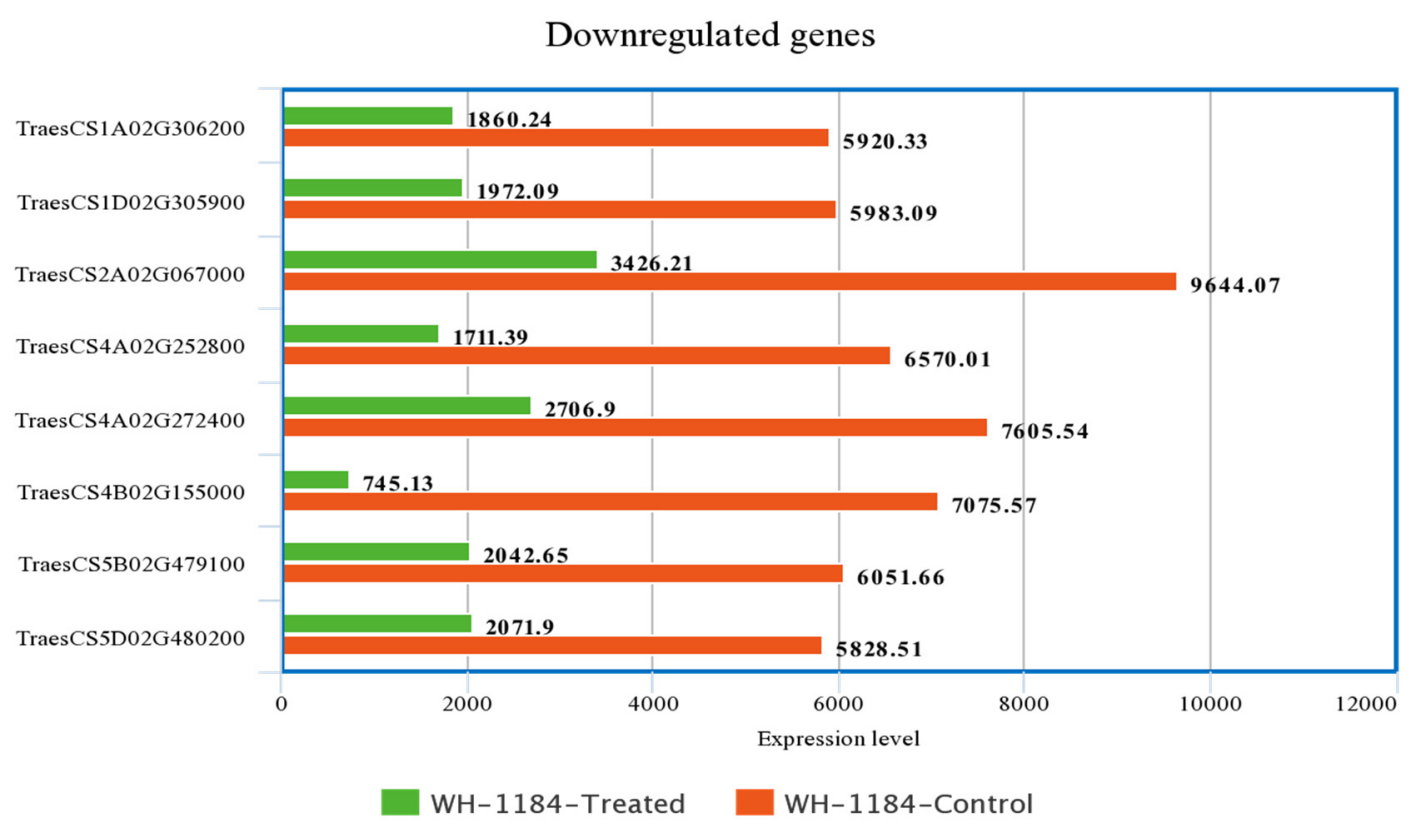
Comparison of highly expressed eight genes under both the conditions.

Genes with absolute log2 fold change ≥ 1 and *p*-value ≤ 0.05 were considered significant (Table 5). The log2 fold change is the log ratio of a genes’s or a transcript’s expression values in two (control verses treated) different conditions. A small *p*-value indicates evidence of differential expression, either overexpression or underexpression. The *p*-value can serve as a probability measure to select differentially expressed genes from a pre-specified significance level (cutoff threshold). A total number of 86,916 genes were tested in WH-1184, under both conditions (control verses treated) and 4260 were found differentially expressed. Out of these, 2308 were up-regulated or induced while 1952 were down regulated genes (Figure 1).

Out of total upregulated and downregulated genes, the expression level of only highly expressed eight genes was taken into consideration for comparison. Upregulated genes which showed thehighest expression level encoded ATP-synt_C domain-containing protein(TraesCS6A02G208600) and putative proteins whereas downregulated genes with greatest expression level that encoded putative proteins, ribulose bisphosphate carboxylase small subunit (RuBisCO small subunit) and Pept_C1 domain-containing protein (TraesCS4A02G252800), were compared to show their expression level in two different conditions. Heat stressed plants exhibited higher expression level of upregulated genes while plants grown under controlled conditions had higher expression level of downregulated gene as compared to heat stressed plants. A description of expression level of regulated genes is presented in Supplementary Materials.

**Figure 2:**
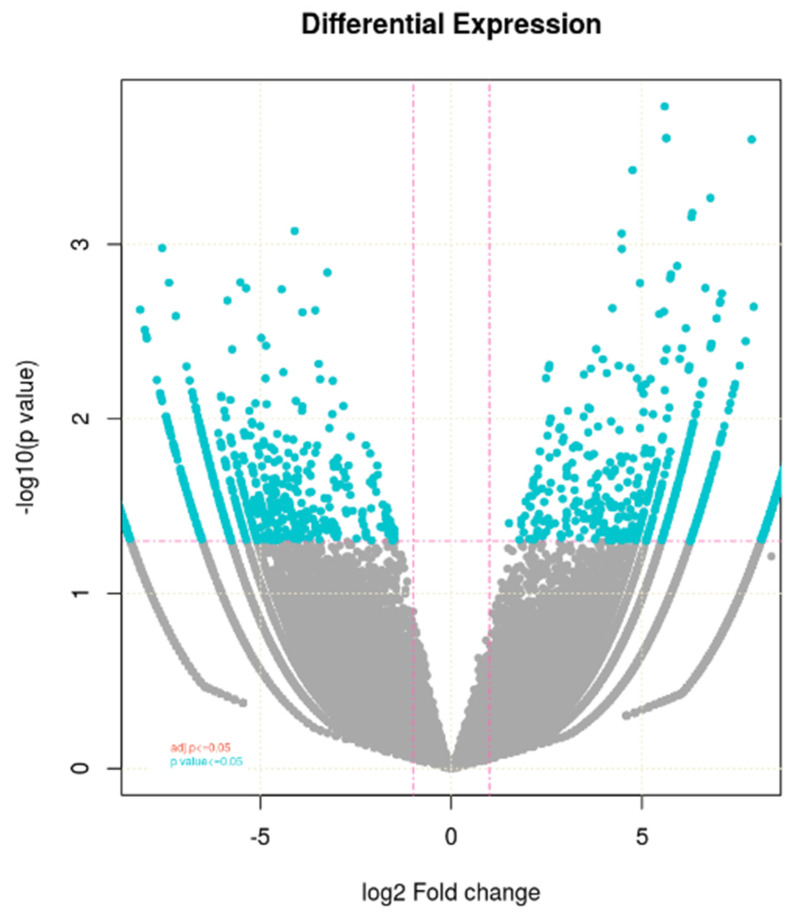
Volcano plot showing differential expressed genes identified between the control and heat treated WH-1184.

The expression profile of differentially expressed genes or to show the how much the genes upregulated and downregulated across the samples is indicated in volcano plots that were constructed as x-axis log_2_ fold change versus y-axis –log10 (*p* value) (Figure 2). Blue dots represent log_2_FC ≥ 1 and *p*-value ≤ 0.05. Pink dotted lines represent absolute log_2_ FC ≥ 1and FDR/adjusted *p*-value ≤ 0.05. This is also used to visualize the biological relevance as well as statistical significance of the genes. So, the values on x-axis represent the biological relevance while y-axis shows statistical significance. The FC value ≥ 1 indicate upregualtion of genes while it is less than 1 shows downregulated genes. One horizontal line represents statistical significance values while two vertical lines represent upregulation and downregulation of genes. The genes between these two vertical lines were discarded for further analysis. This plot indicated that the upregulated genes are greaterin number than the downregulated ones.

### 2.5. Gene Ontology

**Figure 3:**
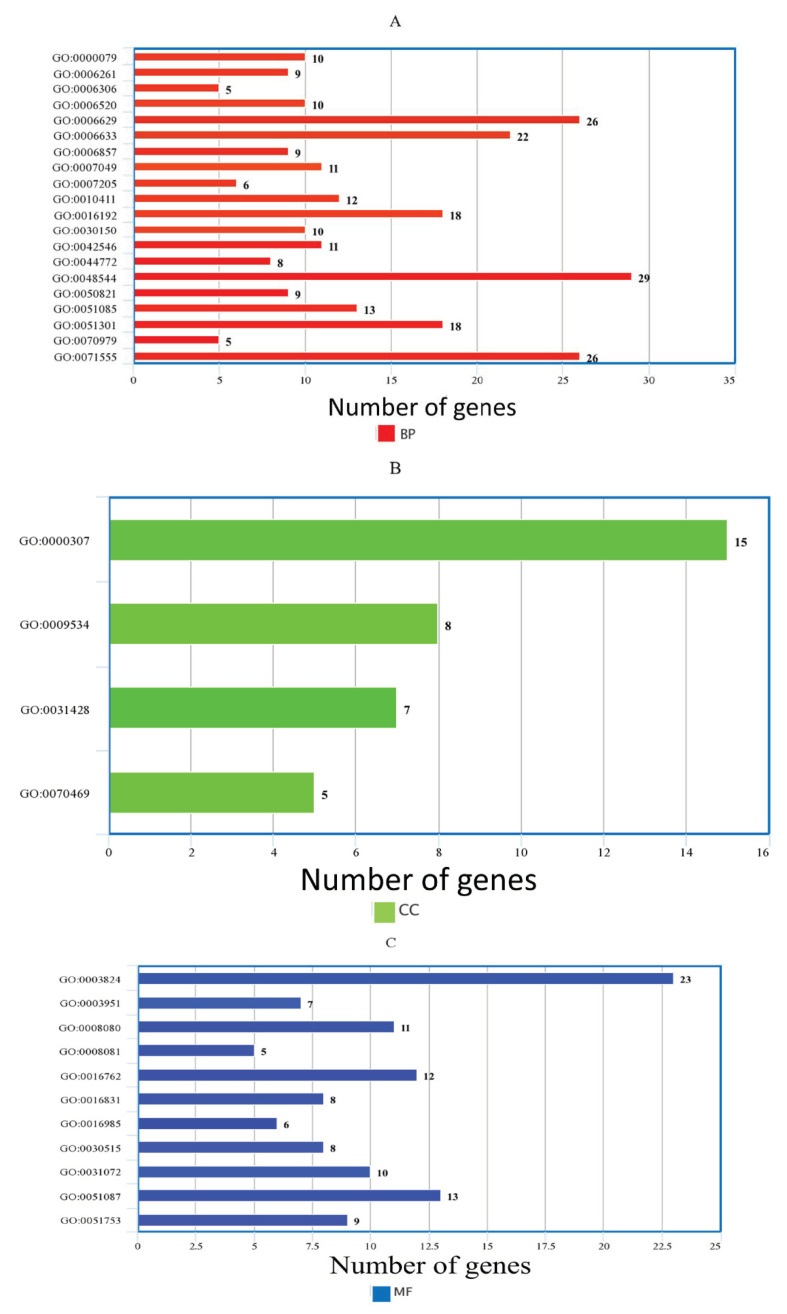
Bar plot of the enriched GO terms, (**A**) Biological processes, (**B**) Cellular components and (**C**) Molecular functions, using significantly expressed genes.

The genes that showed significant differential expression were used to understandmore about the biological pathways encoded by induced and repressed genes, based on Gene Ontology (GO) and pathway enrichment analysis using *ClustetProfiler R package*. The functions of DEGs was analyzed, and 404 genes whose total reads ≥ 5 were considered as significantly enriched genes in GO analysis with *p*-value less than 0.05 for WH-1184. These DEGs are classified into three main domains: biological process (orange color), cellular component (green color) and molecular function (blue color). Out of all significantly expressed (upregulated or downregulated) gene IDs, twenty belonged to biological processes (Figure 3A) and consisted of 257 genes, four were related to cellular components (Figure 3B) with 35 genes, and eleven were associated with molecular functions (Figure 3C) comprising 112 genes (Table 6).

GO annotation results showed that genes related to biological processes encoded regulation of cyclin-dependent protein serine/threonine kinase activity (GO:0000079), DNA-dependent DNA replication (GO:0006261), DNA methylation (GO:0006306), cellular amino acid metabolic process (GO:0006520), lipid metabolic process (GO:0006629), fatty acid biosynthetic process (GO:0006633), oligopeptide transport (GO:0006857), cell cycle (GO:0007049), protein kinase C-activating G protein-coupled receptor signaling pathway (GO:0007205), xyloglucan metabolic process (GO:0010411), vesicle-mediated transport (GO:0016192), protein import into mitochondrial matrix (GO:0030150), protein stabilization (GO:0050821), cell wall biogenesis (GO:0042546), mitotic cell cycle phase transition (GO:0044772),chaperone cofactor-dependent protein refolding (GO:0048544), cell division (GO:0051301), protein K11-linked ubiquitination (GO:0070979) and cell wall organization (GO:0071555). Likewise, genes that belonged to cellular components regulated cyclin-dependent protein kinase holoenzyme complex (GO:0000307), chloroplast thylakoid (GO:0009534), box C/D RNP complex (GO:0031428) and respirasome (GO:0070469) etc. and genes of molecular functions encoded catalytic activity (GO:0003824), NAD+ kinase activity (GO:0003951), N-acetyltransferase activity (GO:0008080), phosphoric diester hydrolase activity (GO:0008081), xyloglucosyl transferase activity (GO:0016762), carboxy-lyase activity (GO:0016831), mannan endo-1,4-beta-mannosidase activity (GO:0016985), snoRNA binding (GO:0030515), heat shock protein binding (GO:0031072), chaperone binding (GO:0051087), and mannan synthase activity (GO:0051753) were significantly expressed under heat stress treated samples. Therefore, it is concluded that in the cellular component, the expression of cell division, cell, and organelle wasprevalent; in molecular function, the terms enzymatic and catalytic activity were dominating; and in biological process, the terms metabolic process and cellular process were dominant. GO:0003824 gene ID belonged to MF (molecular functions) gene category and had maximum expressed genes (23 counts) in WH-1184 which has catalytic activity, followed by 13 counts in GO:0051087 gene that encoded chaperone binding.In BP (biological processes), GO:0048544 gene ID had maximum counts (29) and encoded recognition of pollen, followed by gene IDsGO:0006629 and GO:0071555 with gene count (26) that showed lipid metabolic process and cell wall organization and for CC (cellular components) GO:0000307 gene ID that had maximum gene counts (15) and encoded cyclin-dependent protein kinase holoenzyme complex. Meanwhile, GO:0070469 gene ID showed minimum count (5) as respirasome. A description of differentially expressed genes of WH-1184 is provided in Supplementary Materials (GO:WH1184).

### 2.6. KEGG Ontology Analysis

**Figure 4:**
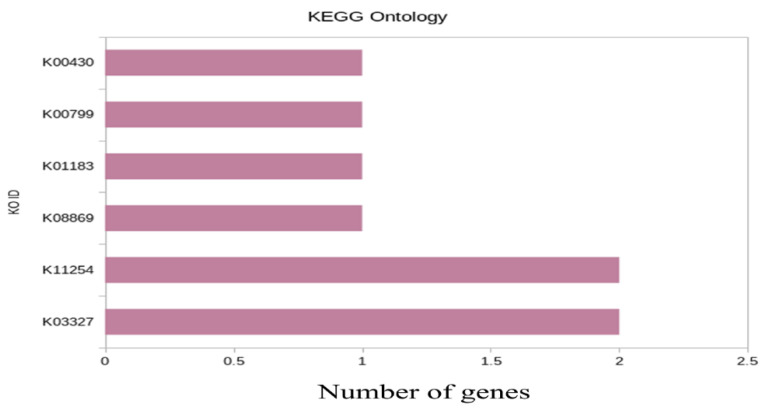
Bar plot of enriched pathway of the significant genes.

KEGG ontology was conductedto further understand the physiological processes of genotypes under heat stress conditions. The statistical enrichment of DEGs in KEGG (Kyoto Encyclopedia of Genes and Genomes) pathways was performed using ClusterProfiler R package. KEGG ontology for WH-1184 was analyzed using the six most significantly expressed genes (Figure 4).

In WH-1184, significant genes (upregulated), K03327 (TraesCS3B02G068700/TraesCS7A02G357000) located on 3B/7A chromosomes, encoded Protein detoxification (Multidrug and toxic compound extrusion protein) and K11254 (TraesCS6B02G288000/TraesCSU02G175900), encoded uncharacterized protein had maximum count values (2) while rest each of four had only one significantly expressed gene (3 upregulated and one downregulated). A description of most significant genes used in KEGG ontology is provided in the Supplementary Materials (KEGGWH1184).

### 2.7. KEGG Pathway

**Figure 5:**
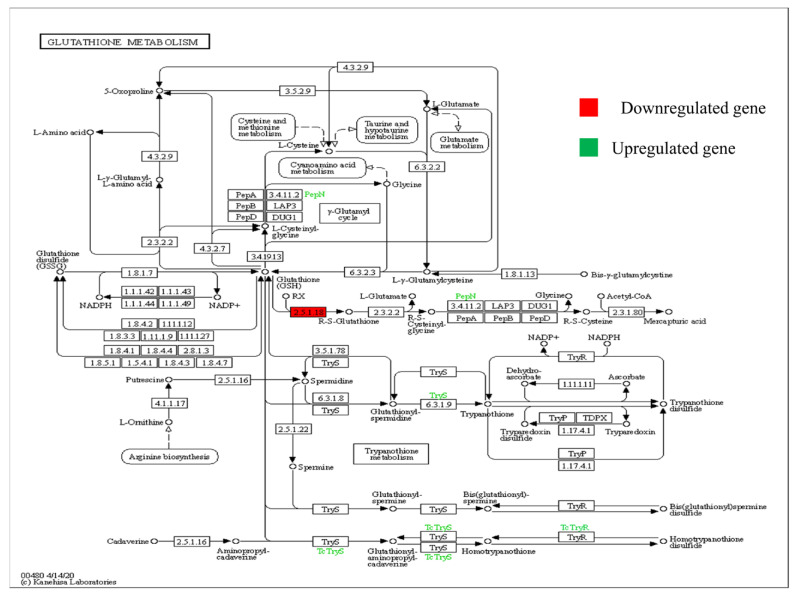
KEGG pathway showing regulation of glutathione-S-transferase enzyme encoded by stress-responsive significantly expressed gene (red colour showing downregulated and green colour is upregulated), *p* < 0.05) under heat stress.

The KEGG pathway is demonstrated with downregulated (red in colour) gene. The pathway represented by Figure 5 shows the glutathion metabolism. The main enzyme that regulatesthispathway is glutathione-S-transferase (GST), and the gene encoding this enzyme is downregulated here while the enzymes shown by green colour are upregulated (PepN and TryS). The downregulation of this gene under heat stressed conditions in WH-1184 genotype causes accumulation of toxic products in plant that may be made the plant sensitive for high temperature. This enzyme (GST) is responsible for synthesis of mercapturic acid which acts as a detoxificant for plants. Trypanothione synthase (TryS 6.3.1.9) is responsible for the production of trypanothione from glutathione spermidine and alanyl amino peptidase (PepN 3.4.11.2) releases N-terminal amino acid.

## 3. Discussion

Climate change and environmental conditions such as heat stress have a significant negative impact on the productivity of the wheat crop worldwide. Heat stress affects over 57% of the area used to grow wheat. Thus, in order to maintain total crop productivity, wheat must be protected from exposure tohigh temperatureduring reproductive (anthesis and post-anthesis) and grain development stages [16,17]. The high temperature causes abiotic stress is a very complicated system that requires the interaction and control of multiple molecular and biochemical processes. Studying the transcription of many important genes and how they are regulated in wheat during the anthesisstage under heat stress is very important. Using RNA-seq-based technology, it is possible to detect important genes, differences in their transcriptional activity, and the self-regulatory attributes with high sensitivity [18].

In this study, the transcriptome profiling of a heat sensitive WH-1184 wheat genotype was studied under control and heat stress conditions to observe the effect of heat stress at transcriptional level also. Kumar et al. [19] evaluate a heat sensitive wheat genotype (HD2329) under control and heat stress conditions. Based on Illumina Novoseq 6000, the differential expression of conserved and novel genes associated with the heat stress wasalso identified. Arenas-M et al. [20] studied wheat genotype Queule-INIA under different conditions and reported a total 29.4 million reads, of which 20.7 million (70.4%) reads were mapped reads. Similarly, Paul et al. [18] removed, 253,046,594 (20.5%) poor quality of rRNA reads and finally considered only 979,825,192 (79.47%) high-quality reads for further analysis (transcriptome assembly). They constructed 2,302,239 transcripts with a minimum read length of 200 bp and GC content of 50.24%. In our study, both samples had 67.40 million of number of reads, of which 11.72 million were mapped, and WH-1184 treated contained higher GC content than WH-1184 control. Zhao et al. [21] suggested that the GC-rich coding region acts as an adaptation and gene regulator that favors efficient translation and also helps in evolution and diversification. A total of 86,916 genes were tested in WH-1184, under both conditions (control versus treated), and 4260 were identified as differentially expressed. Higher number of induced genes was observed in ryegrass with a total of 20,183 differentially expressed genes with higher up-regulated genes than downregulated DEGs during stressed conditions [22]. Wang et al. [23] also found a total of 290,039 transcripts, of which 121,271 unigenes and total differentially expressed geneswere 10.9% (20,198) of total identified unigenes with moreup-regulated than down-regulated genes. The expression level was also compared between both conditions and found that WH-1184 treated had highly expressed upregulated genes while plants under controlled conditions had higher expression of downregulated genes than WH-1184 heat stressed. A compared expression level of genes responsible for heat tolerance suggested that treated plants showed higher expression of these genes than plants grew under controlled conditions [20]. Heat stress, water stress, and combined stresses are also responsible for changes in expression of up-regulated and down-regulated transcripts [24]. Total upregulated and downregulated genes were also indicated by constructed volcano plot. Mishra et al. [25] in their investigation regarding high and low zinc and iron containing wheat genotypes also showed differential gene expression using volcano plot and suggested that red dots represented downregulated genes while green dots for upregulated genes. Yang et al. [26] used a heat map while Kumar et al. [19] used both heat maps and volcano plots for showing the regulation of gene expressions. To analyze functions of DEGs, GO analysis was performedwith classification into three main domains: biological process, cellular component, and molecular function. It was noticed that biological processes had the highest gene IDs while cellular components had lowest. Kumar et al. [19] used GO to observe distribution of identified unigenes in wheat genotype HD 2967 under controlled and stressed conditions and classified into similar three categories. Highest number of identified genes was belonged to biological processes and lowest was in molecular function in two contrasting genotypes [9]. According to Chen and Li [27], in Brachypodium distachyon, highest genes were annotated to biological processes while lowest were in cellular components. Li et al. [28] also used GO analysis in switch grass. Photosynthetic and biosynthetic activities mainly belonged to biological processes while catalytic activities to molecular functions [29]. So, GO explained the functions of different genes under stressed conditions. In this study, heat stress might be disturbed the catalytic activity, cell division enzyme complex, pollen recognition, and cell membrane organization of the heat treated genotype, and this directly and indirectly involves plant sensitivity to heat stress.

KEGG ontology was analyzed for further knowledge and the sixmost differentially expressed genes were used. From this, it was reported that the genes encoding protein detoxification, ABC1 domain-containing protein were upregulated while genes encodingglutathione transferase (GST), peroxidase, and chitinase enzymes were downregulated. Amongthese downregulated enzymes, GST causes reduction of heat stress-related oxidative damages, peroxidase is responsible for higher tolerance for heat or oxidative stress, and chitinase controls the process of plant embryogenesis during abiotic stresses. Meanwhile, upregulated proteins play amain role in detoxification to protect plants from heat stress. Mishra et al. [25] found upregulated peroxidase enzyme under stress conditions and suggested that this protects plant from stressed conditions. The statistical enrichment of DEGs in KEGGpathways was tested using ClusterProfiler R package. Zhao et al. [30] found 1501 differentially expressed genes in drought tolerant genotype and 1623 in drought sensitive for KO terms, whereas out of these 707 DEGs (drought tolerant) were used in 18 KEGG pathways and 212 from drought sensitive for seven pathways and they also found that peroxidase (POD) and (GST) were upregulated in stress tolerant genotype and protect plants from oxidative damage and reduce the accumulation of ROS due to stressful conditions. Noctor et al. [31] also found glutathione (GSH) as a protectant for plant cells from oxidative stress by reducing toxic products, lipid hydroperoxides, and H2O2. Chaich et al. [32] also used the KEGG database to further understand the roles of differentially expressed genes in two genotypes, L-82 and Marvdasht under drought and normal conditions and noticed 47 KEGGS pathways in L-82 treated vs. normal and 46 pathways in Marvdasht treated vs. normal.

## 4. Material and Methods

### 4.1. Plant Material and Stress Treatment

For the experiment, seeds of a wheat genotype WH-1184 were collected from Research farm of Wheat and Barley Section of Department of Genetics & Plant Breeding, CCS Haryana Agricultural University, Hisar. This genotype was selected because it was found heat susceptible at field conditions. The genotype was sown during Rabi season in 2018–2019 under normal and late sown conditions. During the late sown conditions, the maturity stages are coinciding with the terminal heat in this region. Relative stress injury (RSI) and membrane stability index (MSI) were estimated by measuring the electrical conductivity according to the protocol given by Sairam et al. [33]. Statistical analysis for correlation coefficient was done using R software. To further evaluate this at transcriptional level the seeds of the genotype were planted in two different pots, A and B. Each pot contained three plants. Seeded plants of both groups were timely irrigated and proper manual weeding was done. Prior to anthesis stage, the plants were grown in a growth chamber with 22 °C/18 °C (day/night), 12 h/12 h (light/dark) and 60% humidity. During anthesis stage, the plants of pot B were kept in a hot air oven for the stress treatment of high temperature of 42 °C for two hours. The plants of pot A were considered as control while pot B was as heat stressed.

### 4.2. Samples Collection

Flag leaves from main plants of control and heat stressed were collected at anthesis stage in duplicate. As this stage is highly sensitive for the exposure of high temperature, collected samples were immediately transferred to container containing liquid nitrogen (−196 °C) and then frozento −80 °C until further processing of samples; RNA extraction, library preparation, and RNA sequencing [34].

### 4.3. RNA Extraction

RNA was isolated by crushing the samples in powder form in liquid nitrogen and mixed in 500 μL of nucleosol and 200 μL of nuclease free water. After incubating for 15 min, they were centrifuged at high speed and supernatant was collected. To this, equal amount of isopropanol was added and mixed well. The tube contents were loaded onto Zymo Mini Column (Cat. R2062, Zymo Research Kit). RNA pre wash and RNA wash buffers wereused for RNA washing. Finally, RNA was eluted in 20 μL of Nuclease free water.

### 4.4. RNA Integrity and Quantification Check

The RNA quality assessment was done using RNA ScreenTape System (Catalog: 5067–5576, Agilent, Santa Clara, CA, USA) in a 4150 TapeStation System (Catalog: G2992AA, Agilent). The integrity of RNA is determined by RNA integrity number (RINe) assigned by the software. RINe values were measured from 1 to 10, where RINe value 1–5 indicate complete degradation and 5–7 indicate partially degraded RNA and RINe value above 8 indicate good quality RNA. RNA concentration was determined on Qubit^®^ 3.0 Fluorometer (Catalog: Q33216, ThermoFisher Scientific, Waltham, MA, USA) using the Qubit™ RNA BR Assay Kit (Catalog: Q32853, ThermoFisher Scientific).

### 4.5. mRNA Enrichment and Library Preparation

The 250 ng of total RNA was used to enrich the mRNA using NEBNext Poly (A) mRNA magnetic isolation module (Catalog: E7490, New England Biolabs, Ipswich, MA, USA) by following the manufacturers’ instructions. The enriched mRNAs were further taken for the library preparation using the NEBNext^®^ Ultra™ II RNA Library Prep Kit for Illumina (Catalog: E7775S, New England Biolabs). The enriched mRNAs were primed with NEBNext Random Primers and chemically fragmented in a magnesium-based buffer at 94 °C for 10 min in order to get inserts of ~200 nucleotides. The fragmented mRNAs were reverse transcribed to form cDNA and the first strand cDNA reactions were converted to dS DNA. The double stranded cDNA fragments obtained were cleaned up by using 1.8× of AMPure XP beads (Catalog: A63881, 43 Beckman Coulter). The cDNA undergo end repair where in the mix converts the overhangs resulting from fragmentation into blunt ends. To the blunt ended fragments, adenylation was performed by adding single ‘A’ nucleotide to the 3′ ends. To the adenylated fragments, loop adapters were ligated and cleaved with uracil specific excision reagent (USER) enzyme. Size selection was performed according to the manufacturer’s protocol with the addition of AMPure XP beads (Catalog: A63881, Beckman Coulter) aiming for a final library size of 400–600 bp. Furthermore, the cDNA was amplified by 12 cycles of PCR with the addition of NEBNext Ultra II Q5 master mix, and “NEBNext^®^ Multiplex Oligos for Illumina” to facilitate multiplexing while sequencing. The amplified products were then purified using 0.9× AMPure XP beads (Catalog: A63881, Beckman Coulter) and the final DNA library was eluted in 15 µLof 0.1× TE buffer.

### 4.6. Library Quantification and Validation

The library concentration was determined usinga Qubit.3 Fluorometer (Catalog: Q33216, Life Technologies, Carlsbad, CA, USA) using The Qubit dsDNA HS (High Sensitivity) Assay Kit (Catalog: Q32854, ThermoFisher Scientific). Prior to the sample’s measurement, the instrument was calibrated using the two standards provided in the kit. The library quality assessment was performed using Agilent D1000 ScreenTape System (Catalog: 5067–5582, Agilent) in a 4150 TapeStation System (Catalog: G2992AA, Agilent) which is designed for analyzing DNA molecules from 35 to 1000 bp.

### 4.7. Data Analysis

cDNA samples were further processed and sequenced using Illumina Novoseq 6000 and 150 bp paired-end reads were generated. Data quality was checked using FastQC and MultiQC software. The data was checked for base call quality distribution, % bases above Q20, Q30, %GC and sequencing adapter contamination. All the samples have passed the QC threshold (Q20 > 95%). Raw sequence reads were processed to remove adapter sequences and low-quality bases using fastp. The QC passed reads were mapped onto the indexed wheat reference genome (*Triticum_aestivum*.IWGSC) using STAR2 aligner. Differential expression analysis was carried out using the DESeq2 package. Genes with less than 5 reads were removed from further analysis. The read counts were normalized (variance stabilized normalized counts) using DESeq2 and differential enrichment analysis was performed. Genes with absolute log2 fold change ≥ 1 and *p*-value ≤ 0.05 were considered significant. Enrichment analysis for Biological process, Molecular function, Cellular component, and KEGG Pathway was performed using the *ClusterProfiler R package*. Gene Ontology (GO) and pathway terms with multiple test adjusted *p*-value ≤ 0.05 are considered significant. To visualize the GO enrichment results, GO plot R package was used. GO plot package calculates z-score using the following formula,

z score=(up−down)/√count

where *up* is the number of up regulated genes in a GO term and similarly *down* represents number of down regulated genes in the GO term.

## 5. Conclusions

In current investigation, the transcriptome profiling was studied using Illumina Novoseq 6000 for two different conditions and differential expression of genes associated with the heat stress wasalso identified. The compared expression level between both conditions showed that WH-1184 treated had higher expression level of upregulated genes while plants under controlled conditions had higher expression of downregulated genes. These identified DEGs were used for GO to analyze their functions and for KEGG ontology to analyze physiological processes of the genotype. Thus, our findings offer the understanding of differences in transcriptional changes in wheat genotype under controlled and stressed conditions, which aids in resolving the response of molecular processes related to heat tolerance. Finally, we have concluded that the heat tolerance of this better performing genotype (WH-1184) could be enhanced by inducing the expression of suppressed genes as other candidate genes associated with the tolerancecould be identified.

## Figures and Tables

**Table 1:** Description of field parameters; RSI (relative stress injury), MSI (membrane stability index) and GYP (grain yield per plot) under normal and late sown conditions.

Traits	Normal Sown	Late Sown	Percent Reduction	Correlation of GYP in Normal Sown	Correlation of GYP in Late Sown
RSI (%)	16.80	24.59	−46.36	−0.877 **	−0.668 **
MSI (%)	83.22	75.42	9.37	0.876 **	0.668 **
GYP (g)	955.00	584.25	38.82		

** Significant at 0.01 levels of probability.

**Table 2:** Raw sequence data and quality check of raw reads.

Sr. No	Samples	No. of Reads	Data in GBs	GC %	Read Length	%Q20	%Q30
1	WH-1184-Control	57,386,524	8.61	52.5	150	99.75	92.10
2	WH-1184-Treatment	77,403,844	11.61	55.0	150	99.59	86.69

**Table 3:** Read alignment statistics of reads using STAR2 aligner.

Samples	Reads after QC	Mapped Reads	Mapped Reads %	Uniquely Mapped Reads	Uniquely Mapped Reads %	Unmapped Reads	Unmapped Reads %
WH-1184-Control	13,720,122	9,624,294	70.15	5,468,104	39.85	4,095,828	29.85
WH-1184-Treated	18,666,082	13,809,696	73.98	7,637,170	40.91	4,856,386	26.02

**Table 4:** Number of expressed genes in control and treated samples.

Samples	Total Genes	No. of Expressed Genes
WH-1184-Control	107,891	76,012
WH-1184-Treated	107,891	87,694

**Table 5:** Differentially expressed genes (DEGs) in WH-1184 under both the conditions.

Condition	Tested Genes	Differentially Expressed Genes	Up Regulated Genes	Down Regulated Genes
WH-1184-Control_vs_WH-1184-Treated	86,916	4260	2308	1952

**Table 6:** Distribution of the enriched GO terms in three different domains in WH-1184.

Domains	No. of DEGs	DEGs %	No. of Gene IDs
Biological processes	257	63.6%	20
Cellular components	35	8.7%	4
Molecular functions	112	27.7%	11
Total	404		35

## Data Availability

All the data presented in this manuscript itself.

## Supplementary Materials

The following supporting information can be downloaded at: https://www.mdpi.com/article/10.3390/plants11223100/s1, Annexures files GO: WH1184, KEGG: WH1184 and Expression level of regulations showing total number of genes, gene IDs, description of encoded proteins with *p* values and counts.
